# Whole-Genome Sequencing and Transcriptome Analysis of *Ganoderma lucidum* Strain Yw-1-5 Provides New Insights into the Enhanced Effect of Tween80 on Exopolysaccharide Production

**DOI:** 10.3390/jof8101081

**Published:** 2022-10-14

**Authors:** Tuheng Wu, Manjun Cai, Huiping Hu, Chunwei Jiao, Zhi Zhang, Yuanchao Liu, Jian Chen, Chun Xiao, Xiangmin Li, Xiong Gao, Shaodan Chen, Qingping Wu, Yizhen Xie

**Affiliations:** 1 Guangdong Yuewei Edible Fungi Technology Co., Guangzhou 510000, China; 2 School of Bioscience and Bioengineering, South China University of Technology, Guangzhou 510000, China; 3 Guangdong Provincial Key Laboratory of Microbial Safety and Health, State Key Laboratory of Applied Microbiology Southern China, Institute of Microbiology, Guangdong Academy of Sciences, Guangzhou 510000, China

**Keywords:** *Ganoderma lucidum*, genome sequencing, polysaccharides, Tween80, transcriptome, functional gene analysis

## Abstract

*Ganoderma lucidum* is an important medicinal mushroom widely cultured in Asian countries. Exopolysaccharides are bioactive compounds of *G. lucidum* with health benefits. Limited exopolysaccharide content hinders its extraction from *G. lucidum*. The addition of Tween80 had an enhanced effect on *G. lucidum* exopolysaccharide production in submerged fermentation. However, the mechanism of this effect remains unclear. In this study, we report on a high-quality assembly of *G. lucidum* strain yw-1-5 to lay the foundation for further transcriptome analysis. The genome sequence was 58.16 Mb and consisted of 58 scaffolds with an N50 of 4.78 Mb. A total of 13,957 protein-coding genes were annotated and Hi-C data mapped to 12 pseudo-chromosomes. Genes encoding glycosyltransferases and glycoside hydrolases were also obtained. Furthermore, RNA-seq was performed in a Tween80-treated group and control group for revealing the enhanced effect of Tween80 on exopolysaccharide production. In total, 655 genes were identified as differentially expressed, including 341 up-regulated and 314 down-regulated. Further analysis of differentially expressed genes showed that groups of MAPK, amino sugar and nucleotide sugar metabolism, autophagy, ubiquitin-mediated proteolysis, peroxisome, starch and sucrose metabolism, TCA cycle, glycolysis/gluconeogenesis KEGG pathway, glycosyltransferases and glycoside hydrolases played important roles in the enhanced effect of Tween80 on exopolysaccharide production. This work provides a valuable resource for facilitating our understanding of the synthesis of polysaccharides and accelerating the breeding of new strains with a high content of exopolysaccharides.

## 1. Introduction

*Ganoderma lucidum*, commonly known as Lingzhi, is an extremely valuable fungi with many medical properties that has been cultured for a thousand years in Asia. Due to its antioxidant, anti-tumor, anti-inflammatory, antiviral, immune-system strengthening, cognitive-impairment reducing and anti-diabetic bioactivity, polysaccharides from *G. lucidum* have been applied in functional food and pharmaceutical industries [1,2,3,4]. Importantly, *G. lucidum* polysaccharide RF3 had been reported to inhibit the infection of SARS-CoV-2 in animal models [5].

Submerged fermentation of mycelium is an effective approach to obtain *G. lucidum* exopolysaccharides, due to its shorter culture period and seasonal independence [6]. In particular, a polysaccharide isolated from submerged cultured *G. lucidum*, namely GLEP-2, presented enhanced effects on the lymphocyte proliferation [7]. However, according to our previous research, the total content of exopolysaccharides from submerged cultured *G. lucidum* normally ranged from 0.5 g/L to 1 g/L, indicating that content of *G. lucidum* exopolysaccharides cannot meet industrial requirements. Therefore, the improvement of exopolysaccharide production of *G. lucidum* is an important trend. Although many physical approaches such as controlling light [8] can improve exopolysaccharide production, the addition of chemical agents is a more appropriate strategy, due to their low cost and easy separation [9]. Recently, the non-ionic surfactant Tween80, namely polyoxyethylene glycol sorbitan monooleate, has been applied to increase the exopolysaccharide production of mushrooms (e.g., *Lentinus edodes* [10], *Schizophyllum commune* [11]) in submerged fermentation. Our previous work showed that the addition of Tween80 increased the production of *G. lucidum* strain yw-1 exopolysaccharide in submerged fermentation [12] Although Tween80 increased the expression level of key genes, which were involved in the synthesis of exopolysaccharides [12], the mechanism of the enhanced effect of Tween80 on exopolysaccharide production in *G. lucidum* remains unclear.

Studies from microbial exopolysaccharides suggest that the biosynthesis of exopolysaccharides in *G. lucidum* might include precursor production, backbone assembly, side-chain modification, polymerization and export [13]. Current studies of the biosynthesis of polysaccharides in *G. lucidum* have mainly focused on polysaccharide-precursor production. Results of RNAi and over-expression experiments in *G. lucidum* showed that genes encoding phosphoglucomutase (PGM), glucose phosphate isomerase (PGI) and UDP-glucose pyrophosphorylase (UGP) were involved in the biosynthesis of polysaccharides [14,15]. Besides this, glycoside-hydrolase-family proteins and glycosyltransferase-family proteins participate in processes of backbone assembly, side-chain modification and polymerization [16,17].

Since Sanger sequencing technology was created in 1977, sequencing technologies and assembly methods have been developed rapidly. The first report of the *G. lucidum* genome occurred in 2012, using a whole-genome shotgun-sequencing strategy [18]. In 2021, a more complete *G. lucidum* strain Lingjian-2 genome was assembled using the PacBio Sequel system and provided new insights into the genetic mechanism behind its high triterpene content [19]. However, a high-quality genome assembly is needed for the discovery of genetic variation and the breeding of new strains with a high content of bioactive products. Interestingly, a chromosome-level genome of *Lentinula edodes* was assembled using long-read PacBio, Illumina short-read sequencing, and the high-throughput chromatin conformation capture (Hi-C) technique for revealing genetic architecture [20]. Therefore, a third-generation sequencing technique, Illumina short-read sequencing, along with the Hi-C technique, is an effective sequencing strategy for sequencing the *G. lucidum* genome to achieve high-quality genome assembly.

Over the years, a transcriptome based on a next-generation sequencing technique (RNA-seq) has been widely applied to discover the genes for the biosynthesis of polysaccharides. For instance, transcriptomic analysis of three cultivars of *Poria cocos* showed that *malZ*, *galA*, *sord*, *gnl* and *bglX* are key genes related to the polysaccharide biosynthetic pathway [21]. Genes encoding PGM, PGI, UGP, glycoside-hydrolase-family proteins and glycosyltransferase-family proteins were identified by comparative transcriptome analysis of six *Hericium erinaceus* strains as participating in the biosynthesis of polysaccharides [22]. De novo genome sequencing of *Lentinula edodes* and comparative transcriptomic analysis during its developmental stages showed that β-1,3-glucan synthase regulatory genes and β-1,6-glucan synthesis-associated protein (SKN1) genes were involved in polysaccharide synthesis [23]. Therefore, from a technological perspective, the RNA-seq technique can be used in the identification of genes associated with the biosynthesis of exopolysaccharides in *G. lucidum*.

In this study, we first assembled the genome of *G. lucidum* strain yw-1-5 by Oxford Nanopore (ONT), Hi-C and next-generation sequencing (Illumina) technologies, providing genomic information for revealing the biosynthesis of exopolysaccharides in *G. lucidum*. Subsequently the gene-expression changes of *G. lucidum* mycelium with and without the addition of Tween80 were investigated by RNA-seq. This study will provide valuable insights into the mechanism of exopolysaccharide synthesis and Tween80-enhanced *G. lucidum* exopolysaccharide production.

## 2. Materials and Methods

### 2.1. Strain and Mycelium Nucleic Acid Preparation

The *G. lucidum* monokaryon strain yw-1-5 was derived from the dikaryotic strain yw-1 by protoplasting. The strain yw-1-5 was preserved in Guangdong Microbial Culture Collection Center (GDMCC), Institute of Microbiology, Guangdong Academy of Science. The vegetative mycelium of strain yw-1-5, cultured on potato dextrose agar (PDA) medium (200 g potato, 20 g glucose, 20 g agar, 1.5 g KH_2_PO_4_, 1.5 g MgSO_4_, 10 g peptone, 100 mL water) with cellophane for 7 days at 28 °C in darkness, was harvested. The mycelium were then frozen in liquid nitrogen and stored at −80 °C.

Genomic DNA was extracted by QIAGENVR Genomic DNA extraction kit. The concentration and purity of extracted DNA was evaluated by NanoDropTM One UV-Vis spectrophotometer (Thermo Fisher Scientific, Waltham, MA, USA) and then QubitVR 3.0 Fluorometer (Invitrogen, Waltham, MA, USA) was applied to quantify DNA accuracy. Total RNA was extracted from vegetative mycelium of strain yw-1-5. The concentration and purity of total RNA were evaluated by Nanodrop2000. Agilent2100 was then used to determine the RNA integrity number (RIN) value.

### 2.2. De Novo Sequencing and Assembly

Genomic DNA of strain yw-1-5 was used to construct Oxford Nanopore Technologies library with a 20-kb library size according to Oxford Nanopore Technologies standard protocol and the Illumina library with 350-bp library size according to Illumina standard protocol. Sequencing of strain yw-1-5 was performed using Nanopore and Illumina sequel platforms at the Biomarker Technologies Corporation (Beijing, China). The filtered reads were then assembled using NECAT software. The assembled genome was corrected with Illumina data using Pilon software [24]. The high-throughput chromosome conformation capture technique (Hi-C) was used to construct a more accurate genome assembly for *G. lucidum* strain yw-1-5. The vegetative mycelium of strain yw-1-5 was used to construct a Hi-C library and sequenced at Illumina Hiseq platform at Biomarker Technologies Corporation (Beijing, China) according to a previously reported method [25]. Importantly, the construction of the Hi-C libraries are described below. Abstractly, vegetative mycelium cells were cross-linked with formaldehyde and cross-linked cells were subsequently lysed. Chromatin DNA was digested and labeled with biotin-14-dCTP, and then ligated by T4 DNA ligase. The ligated DNA was extracted, purified and sheared into fragments of 300–500 bp. The Blunt-end repair, addition of an A-tail, adaptor addition and purification through biotin-streptavidin-mediated pull-down, PCR amplification were performed, resulting in the construction of the Hi-C libraries.

Finally, these assembly results were integrated into the final assembly. For evaluating the completeness of genome assembly, Illumina data were mapped to assembly sequence by Burrows–Wheeler Aligner (bwa) software [26] and the evaluation with Benchmarking Universal Single-Copy Ortholog (BUSCO) analysis (fungi_odb9 database and BUSCO v2.0 software) [27] was performed.

### 2.3. Genomic Component Analysis and Genome Annotation

Genome component analysis included the prediction of protein-coding genes, non-coding RNAs, pseudogenes and repetitive sequences. For prediction of repetitive sequences, LTR_FINDER v1.05 [28], MITE-Hunter [29], RepeatScout v1.0.5 [30], and PILER-DF v2.4 software [31] were used to construct a repetitive sequence database of the genome of *G. lucidum*. It was then merged with the Repbase database as the final repetitive sequence database. Finally, RepeatMasker v4.0.6 [32] was applied to predict repeat sequences from the *G. lucidum* strain yw-1-5 genome. For the prediction of noncoding RNAs (ncRNAs), transfer RNA (tRNA) genes were predicted with tRNAscan-SE v1.3.1 [33] and Ribosome RNA (rRNA), microRNA (miRNA), and small nucleolar RNA (snoRNA) sequences were predicted with Infernal v1.1 [34] based on the Rfam database. The secondary metabolism gene cluster was predicted by antiSMASH v6.0.0 software [35].

For prediction of protein-coding genes, we used *ab initio* prediction, homologous protein prediction and transcriptome data-prediction methods. We then integrated these three prediction results into the final result. We used Genscan [36], Augustus v2.4 [37], GlimmerHMM v3.0.4 [38], GeneID v1.4 [39], SNAP (2006-07-28) software [40] for de novo prediction. GeMoMa v1.3.1 [41] was used for homologous protein prediction. RNA sequencing was performed using the Illumina HiSeq platform, and standard bioinformatic analysis was performed at the Biomarker Technologies Corporation (Beijing, China). In particular, Hisat2 v2.0.4 [42] and Stringtie v1.2.3 software [43] were used to perform assembly based on transcripts. TransDecoder v2.0 and PASA v2.0.2 software [44] were applied for unigene sequence prediction. Finally we use EVM v1.1.1 [45] to integrate the prediction results and PASA v2.0.2 software was used to modify. For functional annotation of protein-coding genes, predicted protein-coding genes were blasted (e-value: 1 × 10^−5^) against Nr, Swiss-Prot, TrEMBL, KEGG, KOG databases. The GO annotation of protein-coding genes was achieved using Blast2go software [46]. Hmmer software [47] was used for Pfam annotation of protein-coding genes. Furthermore, protein-coding genes were annotated by blast against the Carbohydrate-Active enZYmes Database (CAZy).

### 2.4. Tween80 Treatment and Transcriptome Analysis

The vegetative mycelium of *G. lucidum* strain yw-1-5 was cultured on PDA medium for 7 days at 28 °C in darkness. Fresh mycelium was transferred in 100 mL of modified Martin broth medium (20 g/L glucose, 5 g/L tryptone, 2 g/L yeast extract, 0.5 g/L MgSO_4_ and 1 g/L KH_2_PO_4_) and cultured for 6 days at 28 °C and 120 rpm. Tween80 at a concentration of 2.5% (*v/v*) was added to the medium on day 0. Additionally, reagent-grade Tween80 was purchased from Sangon Biotech Co., Ltd. (Shanghai, China). Mycelium and EPS from the fermentation broth were collected by centrifugation. Mycelium of the Tween80-treated group and control group were then frozen in liquid nitrogen and stored at −80 °C. The content of EPS was assayed by the phenol sulfuric acid method [12]. Data on the content of EPS are presented as the mean ± standard deviation of three replicates. Statistical analysis with GraphPad Prism5 software was performed using Student’s *t*-test.

For revealing the transcriptomic landscape of the Tween80-treated group and control group, total RNA was extracted by RNA Purification Reagent. The concentration and purity of the extracted RNA were evaluated by Nanodrop2000. The quality evaluation was determined by 1.5% agarose gels and Agilent 2100 Bioanalyzer. RNA sequencing libraries were then generated with the Illumina TruseqTM RNA sample prep kit. Next-generation sequencing of these libraries was performed on the Illumina Novaseq 6000 platform at Shanghai Major Biomedical Technology Co., Ltd. (Shanghai, China). Standard bioinformatic analysis was performed using Majorbio Cloud Platform (www.majorbio.com (accessed on 30 September 2022)). Briefly, clean reads were obtained after quality control using fastp v0.19.5 software [48]. The clean reads were then compared with a reference genome using Bowtie2 v2.4.1 software [49]. HISAT2 v2.1.0 [42] and TopHat2 v2.1.1 software [50] were applied to evaluate the results of comparison. All unigenes were functionally annotated based on the NCBI nonredundant protein (Nr) (2021.10), Protein Family (Pfam) v14.6, Swiss-Prot (2021.06), Kyoto Encyclopedia of Genes and Genomes (KEGG) (2021.09), GO (2021.0918) and EggNOG (2020.06) databases. For the quantification of gene-expression levels, FPKMs (fragments per kilobase of exon model per million mapped reads) were calculated using RSEM v1.3.3. The differential expression analysis was performed using DESeq R package v1.24.0 [51]. Genes with an adjusted *p*-value < 0.05 and absolute value of log2 (Fold change) > 1 were defined as differentially expressed genes (DEGs). For the functional annotation of DEGs, gene ontology (GO) enrichment analysis was implemented by GOATOOLS v0.6.5 [52] and the enrichment of differentially expression genes in KEGG pathways was performed by KOBAS v2.1.1 software [53].

### 2.5. Real-Time Quantitative PCR

In total, five differentially expressed genes were chosen to validate these RNA-seq data (Table S15). Fresh mycelium from a Tween80-treated group and control group were collected and ground into a powder with liquid nitrogen. Total RNA was extracted using a HiPure Fungal RNA Mini kit (Magen, Guangzhou, China) according to the manufacturer’s instructions. First-strand cDNA was synthesized by HiScript^®^ III All-in-one RT SuperMix Perfect for qRT-PCR (+gDNA wiper) (Vazyme Biotech, Nanjing, China). The qPCR was performed using a ChamQ Universal SYBR qPCR Master Mix (Vazyme Biotech, China). The 18S rRNA gene was used as the reference gene. The conditions for qPCR were as follows: initiation was conducted at 95 °C for 30 s; followed by 40 cycles at 95 °C for 10 s, 60 °C for 30 s. This experiment was conducted on an Applied Biosystems ABI 7500 (Applied Biosystems, Waltham, MA, USA) containing three technical replicates and three biological replicates. The relative expression of selected DEGs was calculated with the 2^−∆∆CT^ method [54]. The primers used are shown in Table S15.

### 2.6. Data Availability

The genome data of strain yw-1-5 had been submitted to NCBI (National Center for Biotechnology Information, Bethesda, MD, USA) with bioproject ID PRJNA886764. The raw data of the transcriptome generated in this study had deposited in NCBI associated with bioproject ID PRJNA886761.

## 3. Results

### 3.1. Sequencing and De Novo Assembly of Ganoderma lucidum Genome

**Figure 1:**
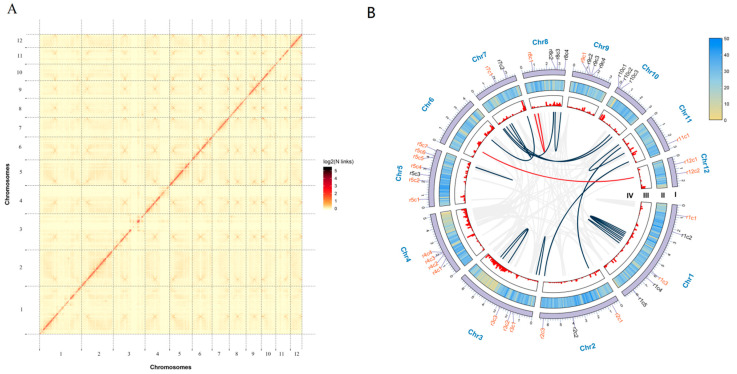
Hi-C interaction heat map and genomic features of the *Ganoderma lucidum* yw-1-5. (**A**), Hi-C interaction heat map of *Ganoderma lucidum* yw-1-5 haploid genome. (**B**), Global view of the yw-1-5 genomic features. The names marked outside the circle were identified as gene clusters and the cluster names in red represent the terpene clusters. The detailed information of these gene clusters was listed in Table S6. Circle I represents the 12 pseudo-chromosomes of *G. lucidum* (Mb). Circle II represents gene density on every pseudo-chromosome. Circle III represents repetitive sequence density on each pseudo-chromosome. Circle IV represents large segmental duplications: regions sharing more than 90% sequence similarity over 10 kb were connected by red lines; those over 5 kb were connected by dark blue lines.

The monokaryon strain yw-1-5, generated from the basidiospore of the dikaryon strain yw-1, was selected for this study (Figures S1 and S2). After filtering for adaptor sequences, low-quality reads, and shorter fragments (<2 kb), 7.23 Gb clean data yielded from the Nanopore sequel platform were obtained and assembled into a 58.16 Mb genome with a GC content of 55.91% for *G. lucidum* yw-1-5 (Table S1 and Table 1). The assembly genome contained 95 contigs (>1 kb), with a contig N50 and N90 of 2.49 Mb and 0.37 Mb, respectively (Table 1). Furthermore, Hi-C technique was used to scaffold the contig assembly and 19.32 million paired reads with 5.78 Gb clean data were obtained (Table S2). Chromosome-scale de novo assemblies of the yw-1-5 genome were achieved by combining the 95 contig sequences and genome-wide chromatin-interaction data sets. A total of 55.52 Mb (95.46%) of the genome sequences were assigned into twelve chromosome groups and there was 100% accuracy in ordering and orienting contigs within chromosome groups (Figure 1A, Table S2). The scaffold N50 value was 4.78 Mb (Table 1). Compared with previous reported *G. lucidum* genomes, the size of yw-1-5 was larger, with fewer scaffold numbers and a larger N50 value (Table S3), indicating the high quality of the genome sequence assembly. In addition, 95.05% of the clean reads from next-generation sequencing could be mapped to the assembly genome (Table S4). The high degree of completeness of the genome was further supported by Benchmarking Universal Single-Copy Ortholog (BUSCO) analysis as 279 BUSCOs (96.21%) were identified and 276 of them were complete (Table S4).

### 3.2. Gene Prediction of Ganoderma lucidum Yw-1-5 Genome

A total of 14,849 genes were mined in yw-1-5 genome based on the homology and de novo methods, with an average gene length of 1824.07 bp, and each predicted gene contained 5.57 exons (Table S5). Using antiSMASH, we identified 38 gene clusters comprising types of terpene, non-ribosomal peptide synthetase cluster (NRPS), NRPS-like fragment, type I polyketide synthase (T1PKS), and beta-lactone containing protease inhibitor (Figure 1B, Table S6). Among these gene clusters, 60.53% are a type of terpene, which is in agreement with the abundant diversity of terpenoid products in *G. lucidum*.

In addition to the protein-encoding gene models, 272 tRNAs were predicted and 10 of them are putative pseudo-genes and the others correspond to the 20 common amino acid and selenium cysteine (SeC) codons. Furthermore, 1,133,073 bases were predicted to encode 319 pseudo-genes and the average length was 3551.95 bp. Moreover, repetitive sequences accounted for 17.45% of the genome, and were more abundant than 8.15% in the haploid *G. lucidum* strain 260125-1 [18]. The most abundant type was the LTR/Gypsy, representing 6.03% of the genome (Table S7).

### 3.3. Functional Annotation of Ganoderma lucidum Yw-1-5 Genome

The genome of *G. lucidum* yw-1-5 had 13,957 genes that could be annotated using a sequence-similarity search in public protein databases (Table S8), including 6699 (45.11%), 3681 (24.79%), 6264 (42.18%), 8599 (57.91%), 6943 (46.76%), 13,768 (92.72%) and 13,921 (93.75%) genes were successfully annotated according to GO, KEGG, KOG, Pfam, Swissprot, TrEMBL and NCBI Nr databases, respectively (Table 2). In addition to these general databases, specific functional databases such as the Carbohydrate-Active enZYmes Database (CAZy), Cytochrome P450 Engineering Database (CYPED) and Transporter Classification Database (TCDB) were used to annotate the function of the identified genes in yw-1-5. A total of 1427 genes could be assigned to these three databases, accounting for 9.61% of the predicted genes of assembled genome (Table 2).

**Figure 2:**
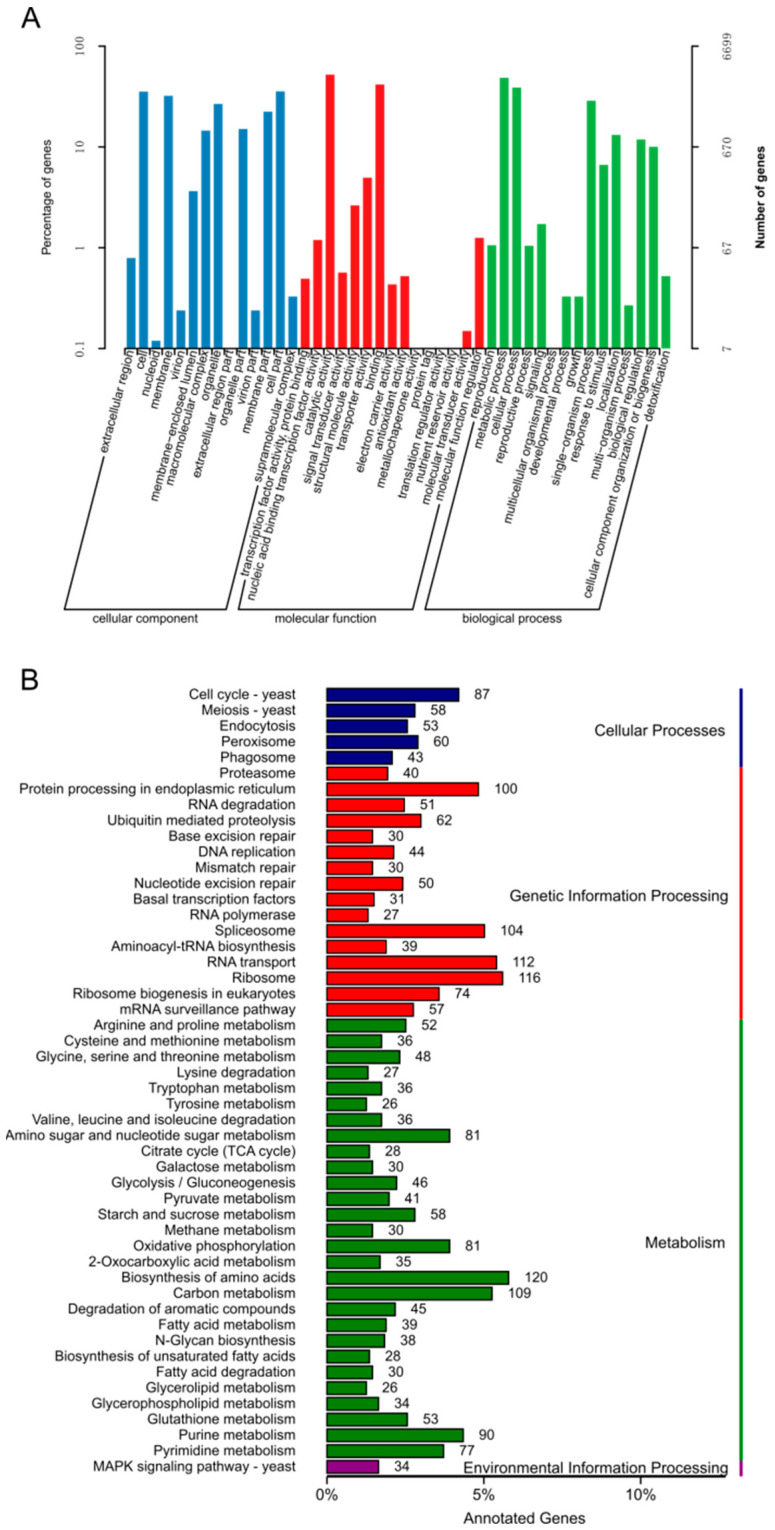
Gene ontology (GO) and Kyoto Encyclopedia of Genes and Genomes (KEGG) functional annotation of the *Ganoderma lucidum* yw-1-5 genome. GO functional enrichment (**A**) and KEGG pathway (**B**) enrichment of putative genes in yw-1-5.

**Figure 3:**
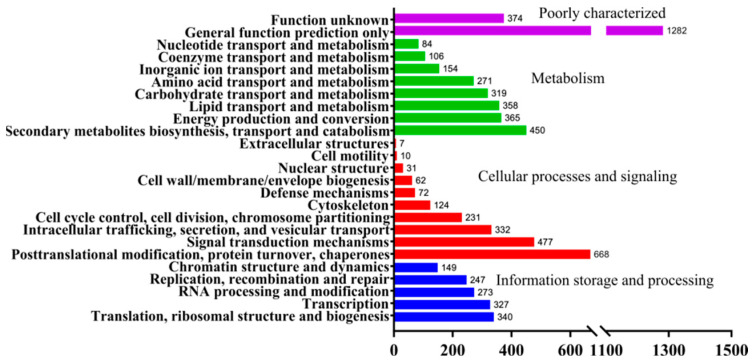
Genes functional classification of yw-1-5 by KOG.

In the GO analysis, genes classified into the biological process were mostly found to participate in the metabolic, cellular and single-organism processes. Within the molecular function category, genes having catalytic and binding activities accounted for 49.1% and 39.2%, respectively (Figure 2). In KOG analysis, most genes were involved in metabolism and 21.36% of them were assigned to secondary metabolite biosynthesis, transport and catabolism, which is well in accordance with the great variety of secondary metabolites in *G. lucidum* (Figure 3). In addition, 2014 genes were found to be involved in cellular processes and signaling, including post-translational modification, protein turnover, chaperones and signal transduction mechanisms. Similar to the KOG annotation results, most genes in the KEGG pathway annotation were involved in metabolism, especially the biosynthesis of amino acids and carbon metabolism, followed by genetic information processing such as RNA transport, ribosome and spliceosome (Figure 2).

It has been reported that cytochrome P450s (CYP450s) are involved in many essential cellular processes and play diverse roles in fungi [55]. In the yw-1-5 genome, we found a total of 685 candidate CYP450 sequences (Table S9), which could be classified into 43 families, and the CYP51 family contained highest counts (135), followed by the CYP620 (88), CYP53 (78) and CYP79 (63) families. The CYP51 family is found across all kingdoms which participate in membrane-ergosterol biosynthesis (sterol 14 alpha-demethylase cytochrome P450 (CYP51), a P450 in all biological kingdoms). The expression profile of one CYP51 member (GL26139) in *G. lucidum* was highly correlated with that of the lanosterol synthase (correlation coefficient (r) = 0.9920). Therefore, we could not exclude the possibility that some of these CYP51 genes in yw-1-5 might be involved in triterpenoid biosynthesis. Furthermore, we found that there are only two CYP51 members have been identified in the haploid *G. lucidum* strain 260125-1 [18], and the *Antrodia cinnamomea* genome contains only one putative CYP51 gene [55]; the increased quantity of CYP51 gene identification and annotation might be a consequence of the improvement in sequencing and annotation pipelines.

### 3.4. Annotation in CAZy Databases

CAZy enzymes (CAZymes) that degrade, modify, or create glycosidic bonds confer wood-decay capability to *G. lucidum*, and play important roles in *G. lucidum* growth and development. Therefore the Carbohydrate-Active enZYmes Database (CAZy) was used to map the genome of *G. lucidum*. In total, 643 candidate CAZymes were identified in the yw-1-5 genome including 101 auxiliary activities (AAs), 15 polysaccharide lyases (PLs), 90 carbohydrate-binding modules (CBMs), 114 carbohydrate esterases (CEs), 313 glycoside hydrolases (GHs) and 83 glycosyl transferases (GTs). The most abundant type was the glycoside hydrolases. For revealing the evolution of CAZymes, a comparison between *G. lucidum* and 10 selected Basidiomycete species was conducted (Table S10). Interestingly, we found that the abundance of CAZymes in yw-1-5 was much more than the 489 in the previous *G. lucidum* genome [19] and was comparable with 614 candidate CAZymes in *Ganoderma leucocontextum* [56].

### 3.5. Genes Involved in Synthesis of Polysaccharides from G. lucidum Genome

Polysaccharides identified from *G. lucidum* fermentation are mainly divided into exopolysaccharides and cell-wall polysaccharides. Generally, the biosynthesis of exopolysaccharides includes the synthesis of nucleotide-activated sugars, the linking and modification of sugar chains, and extracellular export. Exopolysaccharides from *G. lucidum* fermentation are mainly composed of glucose, galactose, mannose, arabinose and rhamnose [7]. Based on the KEGG pathway, the identification of genes associated with the synthesis of nucleotide-activated sugars was performed in our genome data. In the amino sugar and nucleotide sugar metabolism (00520) KEGG pathway, genes related to the synthesis of UDP-Glc, UDG-Gal, GDP-man, GDP-Fuc, GDP-Arb, UDP-GlcNAc, UDP-D-Xyl and UDP-GlcA have been found (Table S11, Figure S3). For instance, the metabolic pathway of the most important nucleotide-activated sugar, UDP-Glc, containing phosphoglucomutase (PGM), glucose phosphate isomerase (PGI) and UDP-glucose pyrophosphorylase (UGP), had been predicted in *G. lucidum* yw-1-5 genome (Table S11). Additionally, for the synthesis of GDP-man, genes encoding mannose-1-phosphate guanylyltransferase (GMPP), phosphomannomutase (PMM) and hexokinase (HK) were also obtained from the *G. lucidum* yw-1-5 genome (Table S11).

Previous studies showed that reactions linking and modifying sugar chains were catalyzed by glycosyltransferases and glycoside hydrolases. Glycosyltransferases are a response for the sugar chains, while glycoside hydrolases are considered as transglycosides in polysaccharide modification. Additionally, the cell wall of fungi mainly consists of chitin and glucans (β-1,3-(1,6)-glucan, β-1,6-glucan). Key enzymes participating in these polysaccharides are glycosyltransferases and glycoside hydrolases. Genes encoding glycosyltransferases and glycoside hydrolases had been predicted through functional annotation in the CAZy database. Because the synthesis of β-1,3-glucan, β-1,6-glucan, chitin had been investigated fully, genes encoding glycosyltransferases and glycoside hydrolases associated with these polysaccharides had been predicted for the *G. lucidum* yw-1-5 genome, as showed in Table S11. In particular, we obtained four genes encoding 1,3-beta-glucan synthase FKS and eight genes encoding beta-glucan synthesis-associated protein KRE6. Finally, there are no reports on the extracellular export of polysaccharides in fungi. New tools should be developed to reveal the mechanism of extracellular export of polysaccharides in *G. lucidum*. Genes annotated in the MAPK (04011) KEGG pathway were also identified in the yw-1-5 genome (see Figure S4).

### 3.6. Transcriptomic Analysis of G. lucidum Mycelium Treated with Tween80

**Figure 4:**
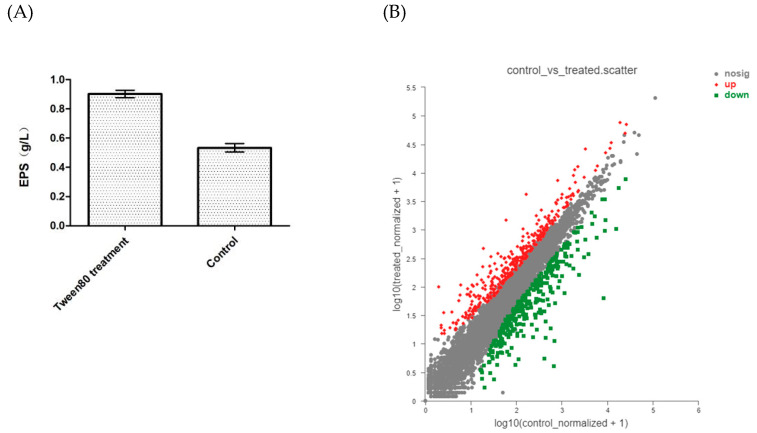
The content of exopolysaccharides (**A**) and the comparison of DEG expression between Tween80-treated group and control group (**B**).

Our previous research showed that a wide range of concentrations of Tween80 had the capacity to increase *G. lucidum* exopolysaccharide production in submerged fermentation [12]. In this study, 2.5% (v/v) Tween80 also increased the production of exopolysaccharide (Figure 4). However, the mechanism of the effect of Tween80 on enhanced exopolysaccharide production in *G. lucidum* is still unknown. Recently, RNA-seq was performed in a Tween80-treated group and control group for the identification of genes associated with this effect (Figure S5). In total, 30.8 Gb clean data were generated from six samples (Tween80-treated group and control group, 3 replicates) (Table S12). There were an average of 5.1 Gb clean data per sample. The Q20 value of each sample was above 97%, the GC of which was above 59%. A total of 10,030 transcripts were obtained after assembly. The transcripts were annotated with the GO, KEGG, NR, Swiss-Prot, Pfam and EggNOG databases for the prediction of possible functions (see Figure S6). In total, 9316 transcripts were annotated and numbers of transcripts annotated in the GO and KEGG were 7303, 3910.

The expression level of each gene was analyzed with the FPKM method (Figure S7). Genes which |log2FC| ≥ 1 and padjust < 0.05 were defined as differentially expressed genes (DEGs). In this study, a total of 655 genes were classified as differentially expressed genes (DEGs), including 341 up-regulated DEGs and 314 down-regulated. The functional annotation of these DEGs with GO and KEGG databases was performed. In GO enrichment analysis, 655 DEGs were categorized into 219 GO terms (Figure S8). Most of the DEGs belonged to the carbohydrate metabolic process, acyl-CoA dehydrogenase activity, interspecies interaction between organisms and carbon–oxygen lyase activity, acting on polysaccharide GO terms. Moreover, KEGG analysis revealed that 655 DEGs were found to map to 78 KEGG pathways (Figure S9). The KEGG pathways with most of the DEGs were peroxisome, steroid biosynthesis and starch, sucrose and galactose metabolism. The results of the KEGG analysis showed that most of the DEGs were categorized into metabolism, suggesting the addition of Tween80 majorly changed the metabolic processes of *G. lucidum* mycelium. Finally, in total, five DEGs were selected to perform qRT-PCR to validate the results obtained from RNA-seq. For each selected DEG, the results of qRT-PCR exhibited similar expression patterns between groups compared with the FPKM values of RNA-seq data, suggesting that the results of RNA-seq were reliable (see Table S17).

### 3.7. Differentially Expressed Genes Involved in the Enhanced Effect of Tween80 on Exopolysaccharide Production

MAPK pathway is essential for the formation and regulation of fungal cell walls [57]. Further analysis of DEGs showed that nine DEGs were mapped in the MAPK signaling pathway—yeast KEGG pathway, including six up-regulated DEGs (*fks2*, *rsp5*, *stt4*, *bmh1,2*, *ptp2,3*, *mkk1,2*) and three down-regulated DEGs (*yck1,2*, *sln1*, *ctt1*). Importantly, gene *fks2* encode glycosyltransferase-family 48 protein, which is a key enzyme of the synthesis of 1,3-β-glucan. These results suggest that these DEGs participated in cell-wall modeling and the regulation of polysaccharide synthesis (Table S13).

There are few exopolysaccharides purified from submerged cultured *G. lucidum*. For instance, GLEP-2 is a exopolysaccharide from submerged cultured *G. lucidum* which is mainly composed of glucose [7]. Besides this, polysaccharides from intracellular parts and the cell wall of *G. lucidum* mycelium include chitin, β-1,3-glucan and β-1,6-glucan. The mechanism of exopolysaccharide synthesis was discussed from three perspectives (nucleotide-activated sugars, linking and modification of sugar chains and extracellular export) based on KEGG annotation. Nucleotide sugars serve as donors for glycosyltransferases that are key enzymes of polysaccharides biosynthesis. In the metabolic pathway of synthetic sugar nucleotides, amino sugar and nucleotide sugar metabolism (00520) KEGG pathway, three genes including *nagB* (encoding glucosamine-6-phosphate deaminase), *nagA* (encoding N-acetyl glucosamine-6-phosphate deacetylase), and gene encoding HEXA_B (hexosaminidase) were up-regulated. In addition, genes encoding chitinase (a glycoside-hydrolase-family 18 protein), xylan 1,4-beta-xylosidase (XYL4), chitin deacetylase, mannose-1-phosphate guanylyltransferase (ManC) were down-regulated (Table S13). In addition, glycosyltransferases and glycoside hydrolases are two important enzymes of polysaccharide synthesis in *G. lucidum*. In this study, we obtained six DEGs encoding glycosyltransferases, including five up-regulated DEGs and one down-regulated DEG. Among them, two DEGs were found to encode β-1,3-glucan synthase and three DEGs participated in the biosynthesis of chitin (Table S14). In addition, eight DEGs encoding glycoside hydrolases were found, along with three up-regulated DEGs and five down-regulated DEGs (Table S14). These results suggest that Tween80 had an effect on the process of exopolysaccharide synthesis in *G. lucidum*.

Previous studies have shown that glucose was consumed more rapidly and the ATP level increased in the Tween80-treated group [12]. They suggested that the carbohydrate-related energy system could participate in the enhanced effect of Tween80 on exopolysaccharide production in *G. lucidum*. In the glycolysis/gluconeogenesis KEGG pathway (00010), *pckA* was found to be up-regulated in the Tween80-treated group. On the other hand, genes encoding pyruvate carboxylase (PYC), phosphoenolpyruvate carboxykinase (PCKA) and fumarate hydratase (FH) from the tricarboxylic acid cycle KEGG pathway (TCA cycle, 00020) were up-regulated. In the starch and sucrose metabolism KEGG pathway (00500), genes encoding glucan 1,3-beta-glucosidase and beta-glucosidase (BglX) were up-regulated, suggesting that the capability for degrading carbohydrates in a medium increased when providing more glucose. In conclusion, starch and sucrose metabolism, TCA cycle and glycolysis/gluconeogenesis KEGG pathways produced energy and materials for polysaccharide synthesis.

Reactive oxygen species (ROS) is an essential part of the abiotic stress response and development in plants [58]. The Tween80 treatment of mycelium in *G. lucidum* led to the accumulation of ROS [12]. The peroxisome KEGG pathway participated in the metabolism of ROS and stress response. In the present study, we found that in the peroxisome KEGG pathway (04146), genes encoding acyl-CoA oxidase (ACOX) and acetyl-CoA acyltransferase 1 (ACAA1) from fatty acid oxidation (β-oxidation) section, PDCR and ABCD from unsaturated fatty acid β-oxidation section, CRAT from other-oxidation PTS1-type section, PEX11 and PMP34 were up-regulated. Moreover, gene-encoding CAT was down-regulated. Interestingly, genes encoding ACOX and ACAA1 were also involved in the fatty acid degradation (00071) KEGG pathway.

Previous studies have shown that fatty acid degradation and the accumulation of ROS induces autophagy [59]. The ubiquitin-mediated proteolysis and autophagy play important roles in cellular homeostasis [60]. In the autophagy-related KEGG pathways autophagy—yeast (04138) and autophagy—other (04139), two genes encoding ATG4 and ATG7 were up-regulated. Additionally, in the KEGG pathway ubiquitin-mediated proteolysis (04120), two up-regulated genes (encoding NEDD4 and ARF-BP1) from “HECT type E3” section and one up-regulated gene (encoding Apc1) from the “target recognizing other subunits” section were found. These results suggest that protein degradation through autophagy and ubiquitin-mediated proteolysis might be an important type of regulation during exopolysaccharide synthesis and Tween80 treatment.

Transcription factors are types of proteins which play important roles in the regulation of transcription through interaction with the promoter region of protein-coding genes [61]. Genes encoding transcription factors associated with the effect of Tween80 on enhanced exopolysaccharide production in *G. lucidum* had never been reported. In this work, we obtained 19 DEGs encoding transcription factors from RNA-seq data, including 14 up-regulated and 5 down-regulated (see Table S16). Among them, 11 DEGs encoding zinc-finger-type transcription factors, especially the HMG-box domain type and fungal-specific type. The DEGs encoding transcription factors need to be investigated by further knock-down and overexpression experiments. Genes related to the synthesis of exopolysaccharides and Tween80 treatment above are listed in Tables S13, S14 and S16.

## 4. Discussion

*G. lucidum* is one of most important medicinal mushrooms in Asia countries. The mycelium of *G. lucidum* yw-1 strain has been used as raw material for Lingzhi products in Guangdong Yuewei Edible Fungi Technology Co., (Guangzhou, China). In addition, polysaccharides have been considered one of main bioactive components of *G. lucidum* for more than twenty years. Due to the medicinal value of *G. lucidum*, we had developed a submerged cultivation technique supplied with Tween80 for enhanced exopolysaccharide production in *G. lucidum* [12]. Since the first report on the genome of *G. lucidum* appeared in 2012, several efforts had been made to achieve a more complete genome [18]. For instance, a more complete reference genome sequence of *G. lucidum* laid the foundation for the breeding of new strains with a high content of bio-active products [19]. Herein, we report a 58.16 Mb reference genome of *G. lucidum* monokaryon strain yw-1-5 containing 14,849 protein-coding genes. Moreover, the strain yw-1-5 genome sequence has been applied to revealing the mechanism of Tween80-enhanced exopolysaccharide synthesis in this study.

Previous studies have reported that long reads, which are generated by the single-molecule Oxford Nanopore technology sequencing platform, have been assembled to a high-quality reference human genome [62]. Besides this, Hi-C is a current approach, based on chromosome conformation capture to detect genome-wide chromatin interaction, and which can be used to widely assist genome assembly [63]. A sequencing strategy combining Oxford Nanopore technology sequence, Illumina data and Hi-C data to generate a chromosome-scale *de novo* assembly of *Lentinus edodes* has been reported [20]. In the present study, we carried out de novo genome sequencing of *G. lucidum* using this sequencing strategy. To our knowledge, the evaluation of assembly by the number of Scaffold and N50 of *G. lucidum* yw-1-5 genome in this work is the best compared with *G. lucidum* genomes from NCBI, proving that Hi-C is an effective method to assist genome assembly in *G. lucidum*. Hi-C data has been mapped to 12 pseudo-chromosomes, whereas the number of chromosomes was thirteen in a previous study [18]. Additional experiments are needed to solve this controversial issue.

Glycosyltransferases are key enzymes which catalyze the formation of glycosidic bonds during the synthesis of polysaccharides [16]. Glycoside hydrolases can degrade, modify and create glycosidic bonds, and are responsible for the modification of sugar chains [17]. In total, 313 genes encoding glycoside hydrolases and 83 glycosyl transferases were obtained from the *G. lucidum* yw-1-5 genome. Genes from the amino sugar and nucleotide sugar metabolism KEGG pathway (00520) had been proved to be involved in the synthesis of nucleotide-activated sugars [64]. Research papers have shown that genes which are involved in the MAPK signaling pathway—yeast KEGG pathway participate in the regulation of exopolysaccharide synthesis [65]. In this study, we also found genes mapped to these two KEGG pathways in the *G. lucidum* genome. Therefore, our *G. lucidum* yw-1-5 genome sequence laid the foundation for researching the synthesis of exopolysaccharides.

Many efforts (e.g., culturing in fine wheat-bran powder [66], the limitation of nitrogen [67], the optimization of pH [6]) have been made to improve exopolysaccharide production in *G. lucidum*. Our previous work showed that the content of exopolysaccharides in *G. lucidum* increased when the mycelium was treated with Tween80 [12], but the mechanism of Tween80-enhanced exopolysaccharide production is unclear. In this research, the transcriptome sequencing of a Tween80-treated group and control group was conducted based on next-generation technology. In total, 655 differentially expressed genes (DEGs) were found in our transcriptome data, including 341 up-regulated DEGs and 314 down-regulated.

Our transcriptomic study showed that six genes in the MAPK signaling pathway—yeast KEGG pathway were found to be up-regulated, including *fks2* and *mkk1,2*. The increase in the content of exopolysaccharides might be due to the up-regulation of *fks2* which encodes β-1,3-glucan synthase, a key enzyme in β-1,3-glucan synthesis. Another piece of research on *Schizophyllum commune* showed that the up-regulation of *fks2* correlated with changes in the content of the exopolysaccharide schizophyllan after Tween80 treatment [11]. Therefore, we proposed that perhaps Tween80 increases exopolysaccharide production through the enhancement of β-1,3-glucan synthesis in *G. lucidum* mycelium. Previous studies have shown that cell-wall integrity kinase (Mkk1,2) was involved in cell-wall integrity and remodeling [68]. By increasing the expression level of *mkk1,2*, Tween80 regulated cell-wall integrity and cell-wall remodeling in *G. lucidum* mycelium.

Nucleotide sugars are blocks for the synthesis of polysaccharides. The amino sugar and nucleotide sugar metabolism (00520) KEGG pathway is involved in the metabolic pathway of synthetic sugar nucleotides. Our transcriptomic results showed that seven genes from this KEGG pathway were differentially expressed. Surprisingly, genes encoding phosphoglucomutase (PGM) and UDP-glucose pyrophosphorylase (UGP) were not differentially expressed. Our previous work showed that genes encoding phosphoglucomutase and UDP-glucose pyrophosphorylase were up-regulated in *G. lucidum* mycelium treated with 0.25% Tween 80 on day 8 [12]. Controversial results in our transcriptome data might be due to the synthesis of nucleotide-activated sugar UDP-Glc in *G. lucidum* mycelium was dynamic during fermentation and Tween80 treatment. In addition, glycosyltransferases and glycoside hydrolases are two key types of enzyme in polysaccharide synthesis in *G. lucidum*. Importantly, we obtained six DEGs encoding glycosyltransferases, two of which encoded β-1,3-glucan synthase. Therefore, Tween80 might increase the content of exopolysaccharide in *G. lucidum* mycelium through the enhancement of β-1,3-glucan synthesis. Three DEGs encoding glycosyltransferases participated in the biosynthesis of chitin were also obtained, suggesting that the change in cell-wall component and cell-wall integrity is adapted to Tween80 treatment. Eight DEGs encoding glycoside hydrolases were identified and will be functionally investigated further.

In *Arabidopsis*, RBOH-mediated ROS facilitated lateral root emergence by promoting the remodeling of the cell wall of overlying parental tissues [69], indicating that ROS are associated with changes in cell-wall components. Surfactant CTAB induced the accumulation of ROS and enhanced mannatide production [70]. Previous studies have reported that the Tween80 treatment of *G. lucidum* mycelium lead to the accumulation of ROS [12]. It is suggested that Tween80-induced ROS might play a specific role in growth, the excretion of exopolysaccharides and cell-wall remodeling, resulting in an increase in the content of exopolysaccharides in *G. lucidum*. In our transcriptome data, seven up-regulated genes in the peroxisome KEGG pathway were achieved and the expression of these genes might contribute to the metabolism of ROS. Above all, Tween80 could induce ROS accumulation through the peroxisome pathway, which might lead to cell-wall remodeling and the excretion of exopolysaccharides. However, proteins linking ROS accumulation with the excretion of exopolysaccharides are needed to investigate this.

ROS-induced autophagy and ubiquitin-mediated proteolysis are two types of protein degradation, participating in cellular homeostasis [60]. ATG4 protease, considered an essential component for the biogenesis of autophagosome, was regulated by reactive oxygen species in *Chlamydomonas reinhardtii* [71]. In this study, one gene encoding ATG4 in the autophagy-related KEGG pathway was up-regulated, indicating that ATG4 protease might be a link of Tween80-induced ROS and autophagy. It then promotes the production of exopolysaccharides. In addition, two up-regulated genes encoding NEDD4 and ARF-BP1 from the ubiquitin-mediated proteolysis KEGG pathway were identified. Other studies have shown that as a post-translational modification, ubiquitination controls a few of the steps in autophagy [71], that is, there is cross-talk between autophagy and ubiquitin-mediated proteolysis. In conclusion, autophagy and ubiquitin-mediated proteolysis might be crucial for the Tween80-enhanced production of exopolysaccharides in *G. lucidum*. Previous studies showed transcription factors such as the blue light receptor–white collar complex (WC-1, WC-2) regulate fruiting-body formation and cell-wall development [72]. We found 19 DEGs encoding transcription factors from RNA-seq data, which might play important roles in Tween80 treatment and exopolysaccharide production. Interestingly, 11 DEGs encode zinc-type transcription factors. These 19 DEGs had not been studied in *G. lucidum* and need to be investigated by functional experiments.

**Figure 5:**
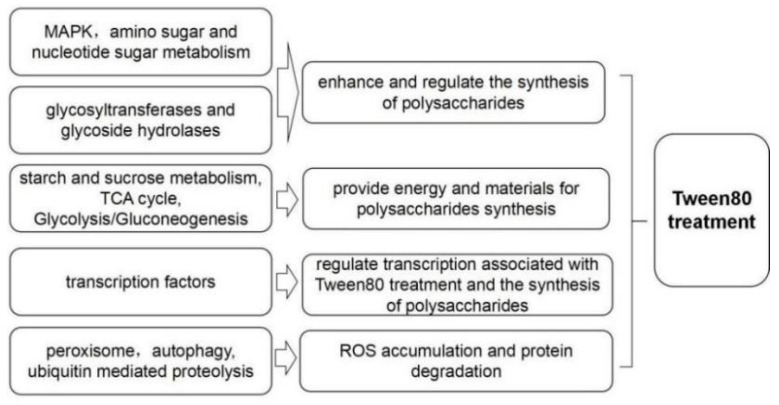
A model of the enhanced effect of Tween80 on exopolysaccharide production.

Finally, according to the results of our genome and transcriptome, we propose a model for explaining the enhanced effect of Tween80 on exopolysaccharide production, as shown in Figure 5. Abstractly, through the regulation of the expression level of genes belonging to KEGG pathways (e.g., the MAPK signaling pathway) and genes encoding transcription factors, glycosyltransferases and glycoside hydrolases, Tween80 enhances exopolysaccharide production in *G. lucidum*. This model should be further investigated by knock-down (e.g., RNAi), knock-out (e.g., CRISPR-Cas9 technology) and over-expression experiments.

## 5. Conclusions

Firstly, the addition of Tween80 under submerged fermentation is an effective approach to increase exopolysaccharide production in *G. lucidum*. We obtained a high quality de novo assembly of *G. lucidum* strain yw-1-5 combining Oxford Nanopore technology sequencing, Illumina data and Hi-C data. The elucidation of this genome provided crucial genomic information to investigate the synthesis of exopolysaccharides. We also obtained genes encoding glycosyltransferases and glycoside hydrolases, which are potential key enzymes of the synthesis of exopolysaccharides. Moreover, our study is the first to show that genes from MAPK, amino sugar and nucleotide sugar metabolisms, autophagy, ubiquitin-mediated proteolysis, peroxisome, starch and sucrose metabolism, TCA cycle and glycolysis/gluconeogenesis KEGG pathways and genes encoding glycosyltransferases and glycoside hydrolases might be involved in the enhanced effect of Tween80 on exopolysaccharide production as revealed by RNA-seq technology. To summarize, this study provides new insights into the synthesis of exopolysaccharides in an important medicinal mushroom, *G. lucidum*, and the addition of Tween80 can be used to enhance exopolysaccharide production under submerged fermentation.

## Figures and Tables

**Table 1:** Statistics for genome assembly.

	Contig	Scaffold
Number	95	58
Total length (bp)	58,157,106	58,160,806
N50 (bp)	2,485,691	4,775,195
N90 (bp)	365,330	3,060,068
Maximum length (bp)	5,247,307	8,872,950
GC content (%)	55.91	55.91

**Table 2:** Functional annotation of deduced proteins by sequence-similarity search.

Annotated Database	Annotated Number	100 ≤ length < 300	Length ≥ 300
GO_Annotation	6699	1752	4827
KEGG_Annotation	3681	1046	2561
KOG_Annotation	6264	1509	4698
Pfam_Annotation	8599	2102	6409
Swissprot_Annotation	6943	1617	5248
TrEMBL_Annotation	13,768	3926	9646
nr_Annotation	13,921	3999	9718
All_Annotated	13,957	4020	9733
CAZy_Annotation	643		
TCDB_Annotation	99		
CYPED_Annotation	685		

## Data Availability

The data that support the findings of this study had deposited in NCBI associated with bioproject PRJNA886764 and PRJNA886761. In addition, data are also available from the corresponding author upon reasonable request. All data generated or analyzed during this study are included in this published article and its supplementary additional files.

## Supplementary Materials

The following supporting information can be downloaded at: https://www.mdpi.com/article/10.3390/jof8101081/s1. Table S1: Statistics of the raw data and clean reads; Table S2: Statistics of the Hi-C assembly; Table S3: Comparisons of *G. lucidum* assembled genome; Table S4: Mapping rate of the Illumina data and Benchmarking Universal Single-Copy Ortholog analysis; Table S5: Statistics of the gene model; Table S6: Gene clusters in the yw-1-5 genome; Table S7: Statistics of the repetitive sequences in the *G. lucidum* genome; Table S8: Statistics of the gene functional annotation in different databases; Table S9: Cytochrome P450s genes in the *G. lucidum* genome. Table S10: The CAZyme spectra were compared between *G. lucidum* and 8 selected Basidiomycete species; Table S11: The putative genes involved in polysaccharide biosynthesis of *G. lucidum*; Table S12: Summary of sequencing and assembly of RNA-seq; Table S13: Putative genes involved in Tween80 treatment obtained from KEGG pathway analysis; Table S14: Genes encoding glycosyltransferase and glycoside hydrolase associated with Tween80 treatment; Table S15: Oligonucleotide primer sets for qRT-PCR; Table S16: Genes encoding transcription factors involved in Tween80 treatment; Table S17: The relative expression levels of selected DEGs. Supplemental Figures, Figure S1: Fruiting body of *G. lucidum* dikaryon strain yw-1; Figure S2: *G. lucidum* dikaryon strain yw-1 and monokaryon strain yw-1-5 grown on PDA plate; Figure S3: Genes annotated in amino sugar and nucleotide sugar metabolism (00520) KEGG pathway from genome; Figure S4: Genes annotated in MAPK (04011) KEGG pathway from genome. Figure S5: Tween80-treated group and control group; Figure S6: Functional annotation and classification of transcripts; Figure S7: The distribution of expression level in Tween80-treated group and control group; Figure S8: Gene ontology analysis of DEGs between Tween80-treated group and control group; Figure S9: KEGG pathway distribution of DEGs between Tween80-treated group and control group.
