# Supplementary material for: Whole-Genome Sequencing and Transcriptome Analysis of Ganoderma lucidum Strain Yw-1-5 Provides New Insights into the Enhanced Effect of Tween80 on Exopolysaccharide Production
Source: J Fungi (Basel). 2022 Oct 14;8(10):1081. doi: 10.3390/jof8101081 (PMC9605614; doi:10.3390/jof8101081)
Supplement: Supplementary file 1 [file jof-08-01081-s001.zip › Supplemental Figures.pdf]

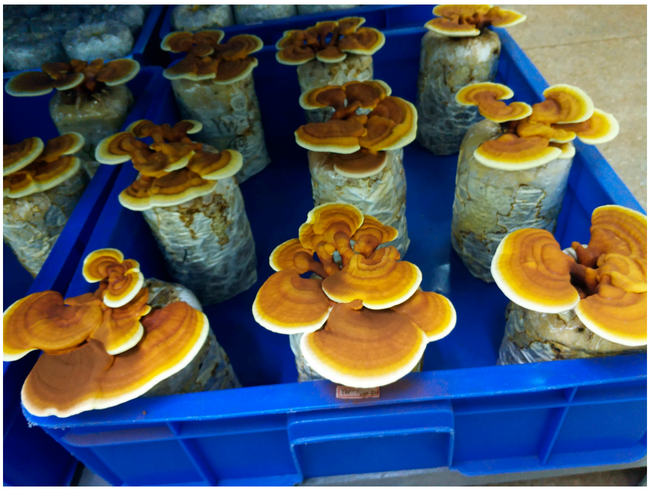

Figure S1. Fruiting body of *Ganoderma lucidum* dikaryon strain yw-1.

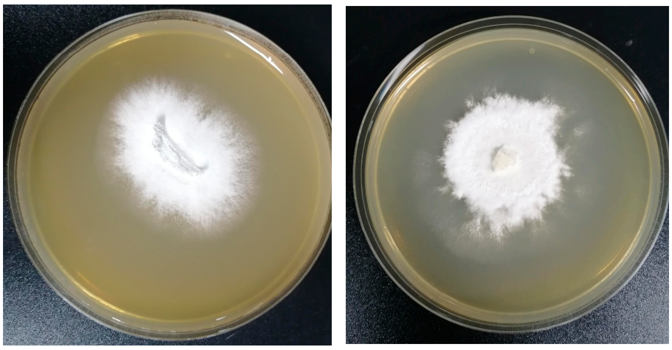

yw-1 yw-1-5

Figure S2. *Ganoderma lucidum* dikaryon strain yw-1 and monokaryon strain yw-1-5 grown on PDA plate

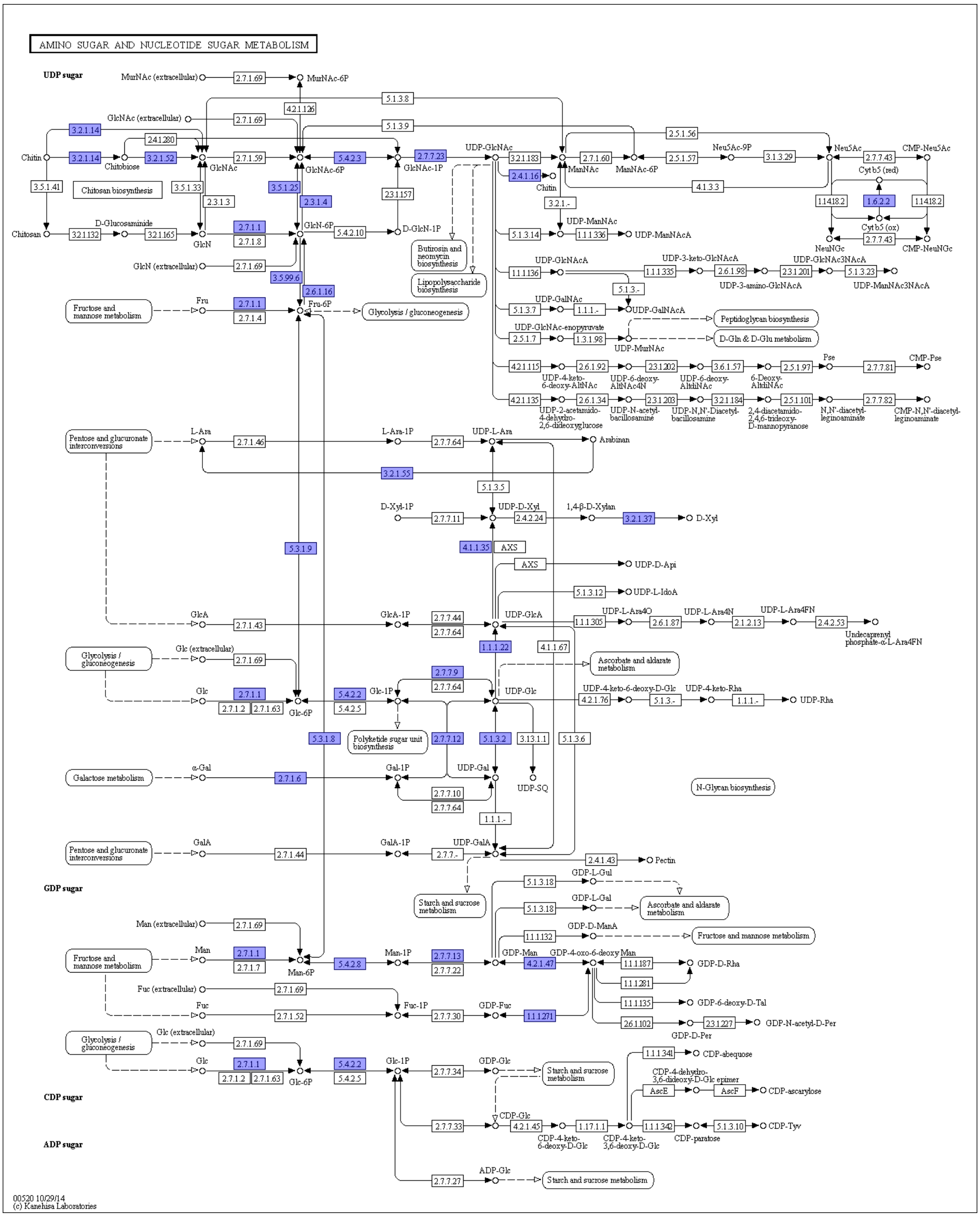

Figure S3. Genes annotated in Amino sugar and nucleotide sugar metabolism (00520) KEGG pathway from genome

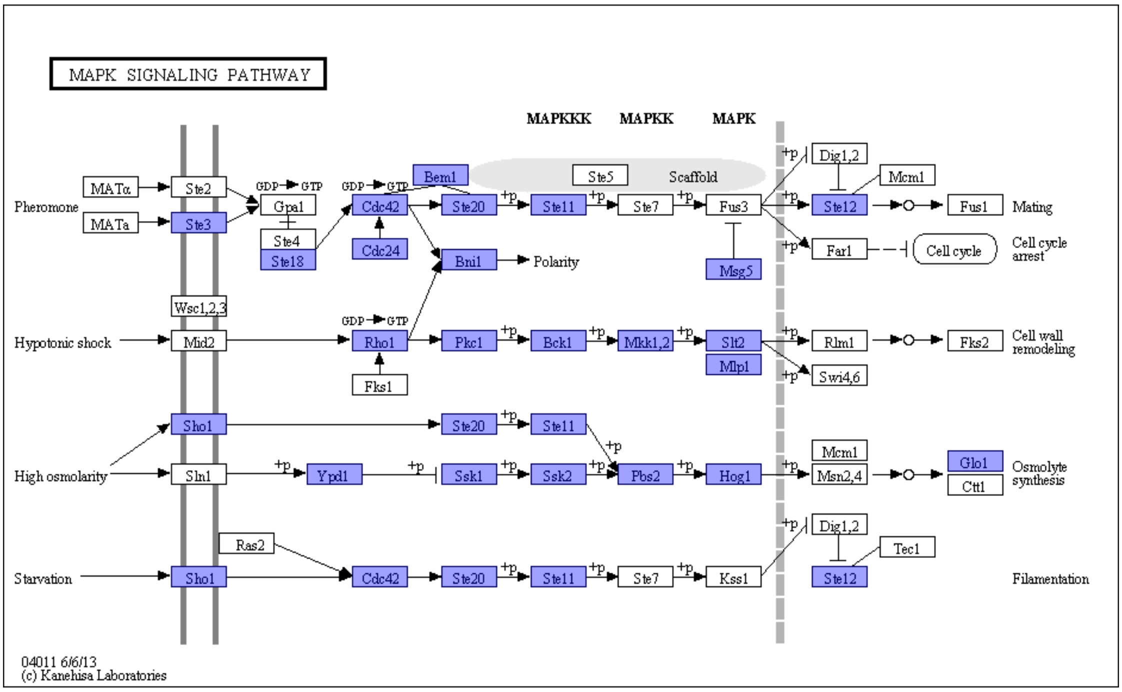

Figure S4. Genes annotated in MAPK (04011) KEGG pathway from genome

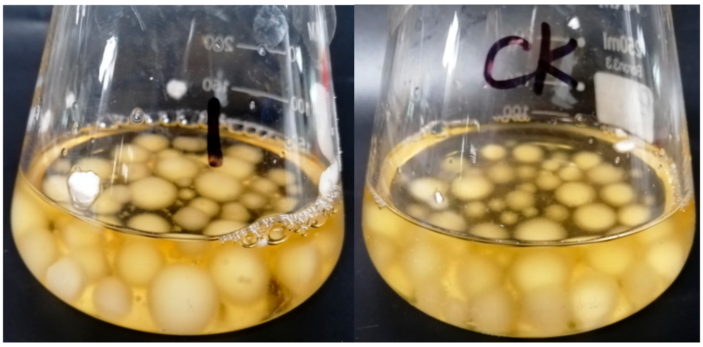

Tween80 treated group control group

Figure S5. Tween80 treated group and control group

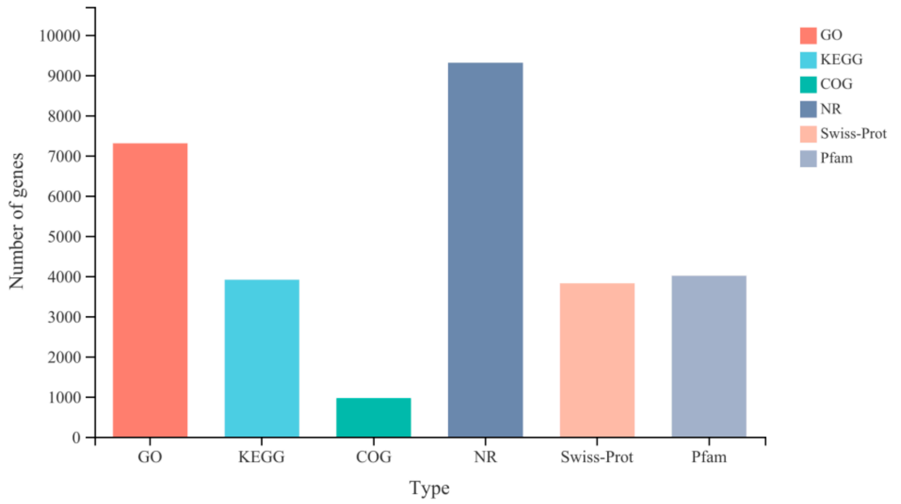

Figure S6. functional annotation and classification of transcripts

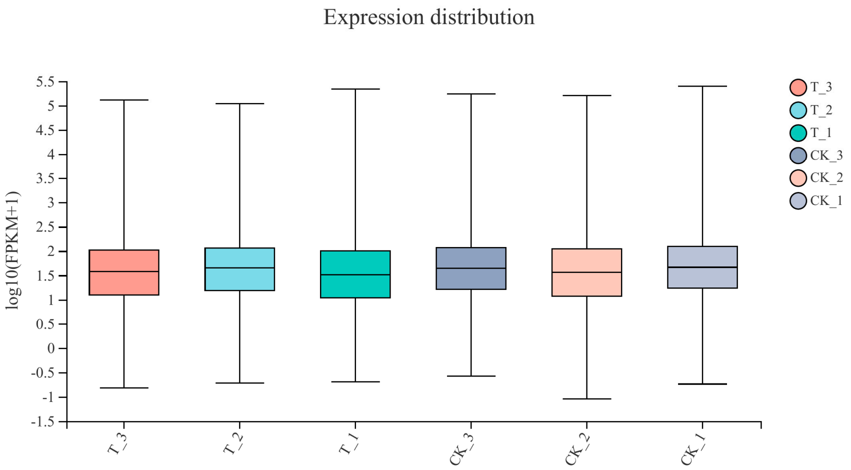

Figure S7. The distribution of expression level in Tween80 treated group and control group.

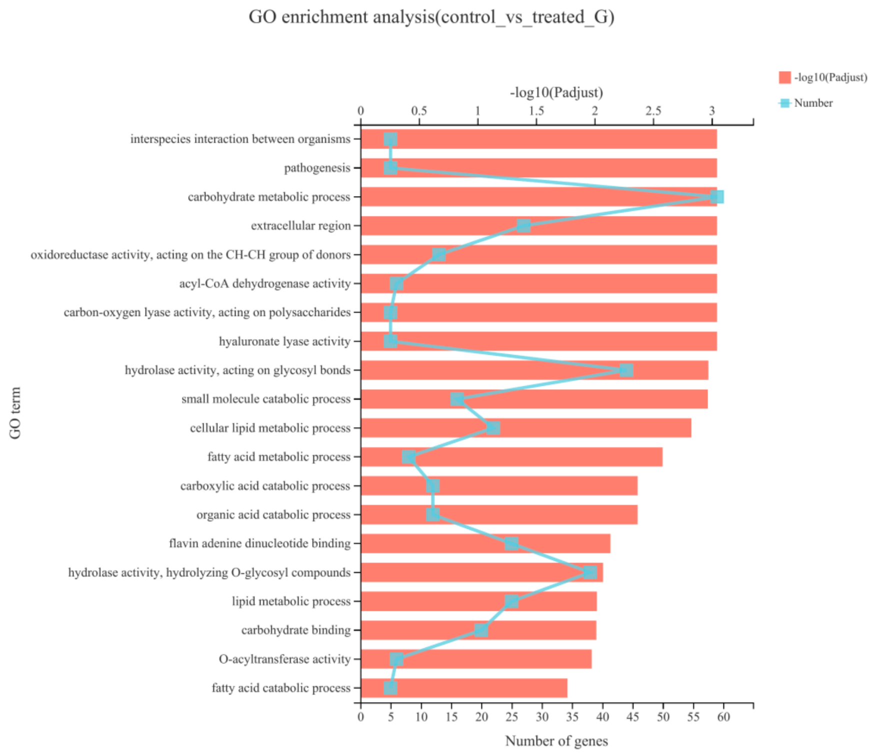

Figure S8. Gene Ontology analysis of DEGs between Tween80 treated group and control group

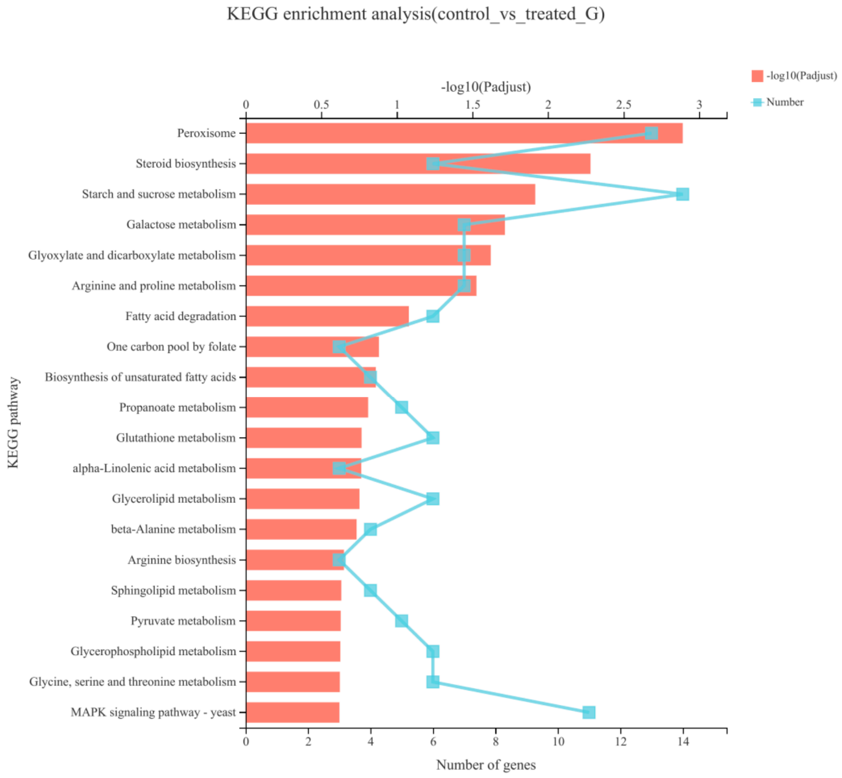

Figure S9. KEGG pathway distribution of DEGs between Tween80 treated group and

control group
